# The Effect of Pollutant Gases on Surfactant Migration in Acrylic Emulsion Films: A Comparative Study and Preliminary Evaluation of Surface Cleaning

**DOI:** 10.3390/polym13121941

**Published:** 2021-06-11

**Authors:** Laura Pagnin, Rita Wiesinger, Ayse Nur Koyun, Manfred Schreiner

**Affiliations:** 1 Academy of Fine Arts Vienna, Institute of Science and Technology in Art, Schillerplatz 3, 1010 Vienna, Austria; r.wiesinger@akbild.ac.at (R.W.); m.schreiner@akbild.ac.at (M.S.); 2 Institute of Materials Chemistry, Technische Universität Wien, Getreidemarkt 9/165, 1060 Vienna, Austria; ayse.koyun@tuwien.ac.at; 3 Institute of Chemical Technologies and Analytics, Technische Universität Wien, Getreidemarkt 9/164, 1060 Vienna, Austria

**Keywords:** acrylic emulsion films, surfactant migration, pollutant gases, 3D microscopy, atomic force microscopy, Raman spectroscopy, FTIR spectroscopy, cleaning treatments

## Abstract

From their first employment in the 1950s, acrylic emulsions have remained widely used as art material today. Although under certain deteriorating conditions they are very stable, if exposed to high humidity and atmospheric pollutant gases, their structural and chemical conformation is strongly affected. Dealing with the resulting surfactant migration, various cleaning treatments were considered over the years. However, their choice remains difficult as they easily alter the acrylic component, especially if in contact with aqueous solutions. The present study focuses on investigating the stability of acrylic emulsion films exposed to accelerated aging by various pollutant gases. Firstly, a comparative analytical study was carried out in order to morphologically (by 3D optical and Atomic Force Microscopy) and chemically (by Raman and Infrared spectroscopy) characterize the reactions and degradation products. Subsequently, two water-based cleaning treatments were tested, and a preliminary evaluation of their cleaning effectiveness was performed. The results show that the reaction of atmospheric gas pollutants with water molecules in moisture leads to acidic reaction products that attack the acrylic matrix and favor the migration of the surfactant to the surface. The effectiveness of cleaning treatments depends on the aging conditions applied, which further lead to different surface morphological changes.

## 1. Introduction

The first water-based acrylic emulsion paint used as an art material was produced in 1954 [1], presenting innovative features such as fast drying time, dilution with water, and flexibility [2]. Since the 1980s, the main chemical composition of acrylic emulsion paints was poly butyl acrylate/methyl methacrylate (p(nBA/MMA)), although there are also variants of contemporary commercial products such as polyethyl acrylate/methyl methacrylate (pEA/MMA). Its easy availability, low cost, and versatility have made acrylic emulsion extremely popular, and indeed, nowadays, contemporary art collections mainly include artworks composed of this binding medium [3]. The drying process of acrylic emulsion paints occurs through a process known as polymeric coalescence. Once the paint has been applied, the acrylic polymer molecules are assembled in droplets suspended in the aqueous phase. During the drying phase, the droplets begin to become closer to each other as water evaporates, eventually forming a continuous film. The degree of coalescence of the polymer can vary depending on the ambient conditions during drying, the glass transition temperature (Tg) of the paint film, the elasticity, the viscosity, and the presence of additives in the mixture that can affect the film porosity [4]. Therefore, the physical properties of dried acrylic emulsion paint can vary according to the distribution and concentration of these components in the mixture, which influences the film formation process.

Specifically, additives allow higher polymer stability in the aqueous phase, the cohesion of the film, and pH stability. Being non-volatile, they also allow a better mixing or thickening of the paint components by remaining within the paint film after coalescence and curing. Therefore, their presence can affect paint aging stability and subsequent preservation treatments such as cleaning [5]. Thus, their identification in painted artworks is important for predicting possible aging behaviors and their reactivity towards the products used during the restoration practices. Several studies [6,7,8] have confirmed that polyethylene oxide (PEO) is the most used additive in acrylic emulsions. Being a non-ionic surfactant, it acts as a wetting, dispersing, and emulsifying agent in paint production. Furthermore, it can stabilize the polymer in aqueous dispersions under different environmental conditions, even in the presence of various pigments. However, it can migrate to the air-film interface [9], resulting in more sensitivity to light exposure. Its accumulation at the interfaces can affect the paint films in terms of mechanical resistance, adhesion to the substrates, permeability, surface gloss, and promoting dirt attraction [10]. 

The migratory behavior of the surfactant derives firstly from the chemical properties of the material, being hygroscopic, and secondly from the environment in which it is exposed. The high percentages of atmospheric water monitored in the outdoor exhibition spaces can favor and increase the surfactant affinity with water. Therefore, hygrometric controls will be necessary in order to monitor this physical-chemical behavior, both for artworks exhibited in outdoor or indoor environments. From a recent study [11], the surfactant migration, particle size, and its distribution were investigated in relation to the exposure of the acrylic films to pollutant gaseous agents commonly present in the ambient atmosphere (SO_2_ and NO_x_). According to the pollutant gas used for accelerated aging, the structural conformation of the particles and their accumulation on the surface changes. For this reason, a part of this study will focus on the morphological and chemical investigation of the different effects that gases cause on polymeric films. The degradation processes of modern art materials, related to the corrosive effect of atmospheric gaseous pollutants, is still a topic of current interest. The stability of outdoor acrylic artworks, such as contemporary murals, paintings on metal, frescoes, and polychrome sculptures, is very unstable as environmental conditions, such as humidity, pollutants, and temperature, are not easily monitored and vary seasonally [12]. Therefore, the results obtained from this investigation could support the conservation and exhibition practices of artworks, also in museum environments. The diagnostic analyses for evaluating their chemical behavior and the continuous environmental monitoring will allow an adequate preventive action of the most sensitive artistic objects [13]. 

As previously mentioned, the surfactant chemical properties favor its surface migration; however, being also soluble in water may be removed by green water-based cleaning methods. The choice of a correct and effective method for cleaning painted surfaces is essential in order to remove degradation deposits and, at the same time, maintain the aesthetic and physical mechanical integrity of the original materials [14]. In the case of acrylic paints, these factors are still being studied. Recent studies [15,16,17] have shown that this material is particularly susceptible to organic solvents and mechanical actions; therefore, further strategies and materials for its cleaning were considered and tested. Water and aqueous solutions have proved to be the most effective systems considering that the effect of pH and conductivity are variables that should not be underestimated for the overall evaluation of the cleaning action. However, this system has some disadvantages in terms of application; in fact, the water directly applied to the surface could cause swelling of the polymeric film with consequent structural and chemical fragility [18,19]. Therefore, the method of applying aqueous solutions is also a widely studied issue. In addition to the evaluation of direct application on surfaces, the use of hydrogels was also widely considered. As a thickening agent that acts as a viscous container for the aqueous solutions it is soaked in, and it can gradually release the water on the deteriorated film with minimum pressure and mechanical action. However, even in this case, some problems were encountered, such as the irregular cleaning effect on the films and the presence of gel residues after application [20]. However, it is important to consider that other variables can also limit the cleaning effect of acrylic works. One of these is the particulate matter (PM), which is a risk for painted surfaces both because its deteriorating effect changes according to the art material (being composed of a mixture of solid and liquid particles suspended in the air), and because its accumulation on surfaces can alter the morphology of the material and compromise the cleaning operations favoring surface abrasion [21]. Another factor that can affect both degradation processes and cleaning practices is the growth of microorganisms—including algae, fungi, bacteria and even lichen—on painted surfaces [22]. In indoor environments, their expansion is influenced by heating, air conditioning, humidifiers/dehumidifiers, and human activities which, however, are more controlled factors than in an outdoor environment. In fact, higher water availability and light exposure might favor the growth of microorganisms depending on the climate of the environment and the exposition of the painted surfaces. Furthermore, additional elements can stimulate (presence of NO_2_ in the urban atmosphere, availability of carbon sources in dust and dirt) or inhibit (metals present in the pigments, SO_2_ from the atmosphere) their expansion on painted surfaces. These factors can make the assessment of degradation processes and subsequent cleaning practices more challenging to monitor and evaluate [23]. The experiments in this study are based on the effect of non-biological factors, and the study of biodeterioration will be a next step in order to achieve a more comprehensive understanding about aging of paint coatings in the environment.

The aims of this study focused on two main objectives: the first is to understand the degradation behaviors observed for pure acrylic films when exposed to various gaseous pollutants. The detected degradation products will be investigated firstly by microscopic and topographic observations. Subsequently, the results will be implemented by the chemical information obtained from the qualitative and semi-quantitative spectroscopic analyses. The focus is to identify the most harmful pollutant gas for acrylic paints and understand which factors increase or mitigate the deteriorating effect. The second objective is to perform a preliminary evaluation of two cleaning systems commonly used in restoration practices. They, having a different impact on acrylic surfaces, will have different cleaning effects, even according to the different pollutants used for accelerated aging. This evaluation represents a precursor study to subsequent innovative cleaning methods for acrylic paints that can be tested and compared to the results introduced here. The diagnostic analysis presented can be used to: support the prevention of the degradation of acrylic materials, develop more sophisticated environmental sensors for monitoring these pollutants (especially in indoor museum environments), and, finally, implement the knowledge related to conservation and restoration practices of acrylic artwork.

## 2. Experimental

### 2.1. Sample Preparation

Several mock-ups were prepared using a pure acrylic emulsion Plextol^®^ D498 (Kremer Pigmente, Aichstetten, Germany). The fresh films were cast on glass slides with a wet film thickness of 150 μm using the so-called doctor-blade procedure [24]. The samples were dried at ambient conditions (approx. 22 °C and RH 30%) for three weeks. 

### 2.2. Weathering Experiments

The artificial gas aging was carried out in a chamber (Bel-Art™SP Scienceware™) made of a co-polyester glass (Purastar^®^), including gas in- and outlets with a total volume of 30 cm^3^. The desired concentration of corrosive gas is generated by humidifying synthetic air 5.0 (Messer, Gumpoldskirchen, Austria) using double-distilled water and subsequently mixing it with the selected gas. The chamber was continuously flushed with the gas mixture with a gas flow rate of 100 L/h. The relative humidity (RH) content chosen is 80% for a total exposure time of 168 h. The gas concentration values were selected according to the annual report released by the European Environmental Agency for air quality monitoring (see Table 1) in order to reproduce a long-term gas aging [25]. The samples were aged with gaseous pollutants representing the main and most harmful corrosive gases at the atmospheric level, namely hydrogen sulfide (H_2_S), sulfur dioxide (SO_2_), nitrogen oxide (NO_x_), and ozone (O_3_). In order to better understand the effects of aging on the polymeric films, a set of samples was aged only at RH 80%.

### 2.3. Cleaning Methods

In this study, cleaning tests were performed on pure acrylic samples once artificially aged. Considering the previously mentioned studies, two cleaning systems were chosen, allowing cross-checking and evaluation of results by selected techniques. 

The first test was by cotton swab rolled, a common cleaning method still used for the conservation of acrylic artworks [26]. Cleaning tests were performed using commercial cotton swabs made from pure pharmaceutical cotton. The cotton swabs were immersed in slightly acidic distilled water (pH around 5–6.5) with a conductivity of around 2–4 µS/cm (pH meter *LAQUAtwin pH33*^®^ and conductivity meter *EC33*^®^, Horiba, respectively). The monitoring of pH and conductivity values is important as they are fundamental parameters for a correct surface cleaning action. The acrylic binders have acidic components in commercial products, thus, chemically susceptible to ionization/dissociation reactions already at pH 6. Therefore, an acceptable pH value is around 5–6 [27]. The cotton swab was used on the surface by applying controlled pressure for an application time of 3 min.

The second cleaning test was performed using a hydrogel system called Nanorestore Gel^®^ Dry [28]. It is a water-based chemical gel developed by the Research Center for Colloids and Nanosciences (CSGI). Specifically, it is composed of poly(2-hydroxyethyl-methacrylate) p(HEMA)/poly (vinylpyrrolidone) (PVP), a hydrophilic polymeric film with high water retention properties and mechanical strength, designed for cleaning highly water-sensitive painting surfaces [29,30]. Its characteristics allow a cleaning action limited to the gel–paint interface. The aqueous solution/solvent is gradually released on the surface, reducing the impact and possible damage to the polymeric film given by the water. Using distilled water to carry out the cleaning test, the specific hydrogel chosen was Nanorestore Gel^®^—Medium Water Retention (MWR), as it is suitable for polymeric surfaces and the removal of water-soluble residues. As indicated by the product technical data sheet [31], it was immersed in distilled water for 12 h. Once the absorption phase was completed, the excess water was removed by placing each side of the hydrogel on absorbent paper for 1–2 s. Subsequently, it was placed on the aged acrylic surface and left to act for 3 min. For both tests, the distilled water used and the time of application was the same allowing a more accurate evaluation of the resulting data. Tests were conducted on all aged samples.

### 2.4. Optical 3D Microscopy 

A Keyence VHX-6000 microscope (Keyence, Osaka, Japan) was employed to scan each sample surface. Three-dimensional morphological images were recorded using a VH-Z100 objective (1000×), obtaining a depth profile of 10 μm (pitch scans every 2 μm). The microscope is equipped with a LED light source (5700 K). The lighting selected was partial coaxial for observations in a partial dark field to emphasize height differences. The pictures acquired were processed by using the free version ImageJ software [32]. This program is usually applied to determine selected areas with a particular shape or a specific color range. This study used this method to obtain histograms representing the distribution and average size of the surfactant particles according to the different gas exposures. Through different image processing steps, the results were obtained as follows. Firstly, the image was converted into an 8-bit grayscale image. In this way, 256 intensity graduations (shade of grey, 0 black, and 256 white) were obtained and were assigned to each pixel. Subsequently, all pixels were separated with intensity graduation in a specific range according to the “thresholding grayscale” function. These pixels formed a unique subset of the image. Finally, the grayscale image was converted into a binary image by defining a grayscale cut-off point. Grayscale values below the cut-off become black (surfactant particles), and those above this value become white (background). The area value for each surfactant particle was obtained from this image processing, and subsequently, the from diameter value useful for the particle size distribution histograms (by using Origin software).

### 2.5. Atomic Force Microscopy (AFM) Combined with Raman Spectroscopy 

Images were obtained using an Atomic Force Microscope WITec Alpha RSA+ (WITec, Ulm, Germany) in tapping mode. The cantilever tip is a WITec arrow reflex-coated FM (AC), spring constant k at 2.8 N/m, with a resonance frequency of 75 kHz, and lateral resolution of down to 1 nm and depth resolution of <0.3 nm. It allows a stereometric analysis obtaining the 3D surface texture of reference and aged acrylic samples. All scans were collected over a 50 × 50 µm^2^ area (512 lines per image). The surface images of films were evaluated using the software WITec Project FIVE 5.1. The data discussed concern the topographies obtained and the respective roughness values (Sa) compared between the reference and aged samples. For an accurate and reproducible evaluation of the topographic results, three different surface areas of the samples were scanned and the surfactant roughness and particle size values were averaged. Statistical data deriving from the evaluation of AFM topographies (average particle size (μ), standard deviation (σ), and correlation coefficient (R)) were obtained using the software Project FIVE 5.1 (WITec, Germany). Specifically, the topographic scans were color line corrected by slope substraction and subsequently the statistical values were obtained in the “image histogram and statistics” section. Atomic force microscopy (AFM) was combined with Raman spectroscopy, using a confocal micro-Raman system (WITec Alpha RSA+). Measurements were performed using 532 nm excitation radiation with a real output laser power of 42 mW, integration time 0.06 s, and time/line 9 s. The sample surfaces were observed with the Zeiss objective 20×, and the scanned areas (20 × 20 µm^2^) were analysed using a camera connected to the microscope. The acquisition of the spectra and their evaluation was performed with the WITec Project 5.1 software. The spectra obtained for the three scanned areas were averaged, baseline corrected, and vector normalized in order to obtain a more reliable chemical mapping of specific Raman bands. 

### 2.6. Attenuated Total Reflection Fourier Transform Infrared Spectroscopy (ATR-FTIR) 

A LUMOS Microscope (Bruker Optics, Ettlingen, Germany) with a germanium crystal was employed for the ATR-FTIR investigations. The instrument is equipped with a photoconductive cooled MCT detector. On each sample, five measuring spots were acquired in the spectral range between 4000 and 480 cm^−1^ performing 64 scans at a resolution of 4 cm^−1^. The resulting spectra were collected and evaluated by the software OPUS^®^ 8.0 (Bruker Optics, Germany). For the qualitative and semi-quantitative analysis, the spectra were averaged, baseline corrected, and vector normalized. Subsequently, the main absorbance bands of the surfactant were integrated. As shown in a previous study [33], selecting specific bands for the semi-quantitative evaluation will allow more reliable data to be obtained, in order to better investigate the chemical changes after aging. In Table S1, the specific surfactant absorbance bands integrated for the semi-quantitative evaluation are listed. For the chemical mapping, the total mapped area had a dimension of 1.0 × 1.5 mm^2^; six measuring spots along the *x*-axis (optical aperture approx. 0.2 mm) and six spots along the *y*-axis (optical aperture approx. 0.1 mm) were collected for a total of 36 spots. Each chemical mapping experiment was carried out in three different areas of the samples.

## 3. Results and Discussion

### 3.1. Three-Dimensional Optical Microscopy

#### 3.1.1. After Aging

**Figure 1:**
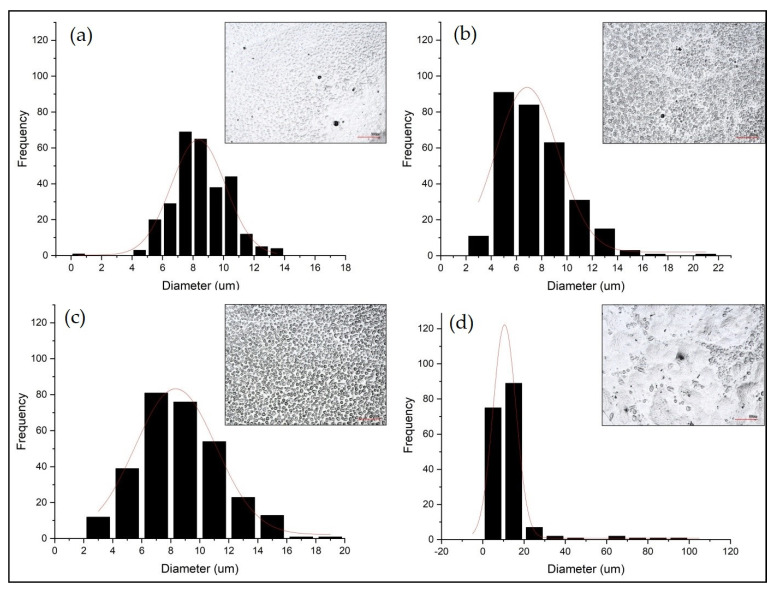
Histograms and Gaussian functions resulting from 3D image processing (top right image in each graph): samples aged at RH 80% with (**a**) H_2_S, (**b**) SO_2_, (**c**) O_3_, and (**d**) NO_x_.

The observations using 3D optical microscopy allowed the morphological changes observed on the surface of the acrylic samples after gas aging to be evaluated. From a first investigation, with all four pollutant gases, it is possible to observe a homogeneous opacification of the surfaces. Furthermore, even with a magnification of 200×, the presence of some surface particles is noted. However, depending on the gas used for accelerated aging, a different feature is observed. With H_2_S, SO_2_, and O_3_, they have a relatively uniform rounded shape and are well grouped together. On the other hand, with NO_x,_ they are less homogeneous, and their size varies. The acrylic sample aged exclusively with RH 80% shows some morphological changes related to the acrylic matrix; these particles are not so evident for a reliable 3D microscopic investigation. For a more detailed understanding of their different superficial migration levels, the evaluation of the particle size distribution and their average size was performed. In the present study, the particle size distribution was represented by histograms resulting from the diameter average of the particles and their frequency on the surface (Figure 1). For all four samples, the particle size distribution trend is described as a Gaussian function. The results, summarized in Table 2, are expressed as correlation coefficient (R), average particle size (μ), and standard deviation (σ).

From Figure 1, different distributions and sizes of the particle are observed. In general, after aging with H_2_S and SO_2_, they appear more cohesive. Their average particle size does not vary significantly, resulting in a well-distributed cluster over the entire surface. In addition, their migration after aging with O_3_ presents a distribution similar to the previous ones; however, the particles are less similar and homogeneous in size. Finally, the most evident morphological changes are observed after aging with NO_x_ (Figure 1d). The average particle size value is very high, indicating that the particles are unevenly distributed and at different sizes on the surface. From these preliminary results, it is already possible to observe the different impacts of the pollutant gases on the polymeric film. Through subsequent AFM results, it will be possible to investigate their identification and effects in more detail.

#### 3.1.2. After Cleaning

As reported in the literature [34,35], one of the main causes of deterioration in contemporary artworks is the surface dirt and the accumulation of pollutants. In the specific case of acrylic binders, the pollutant agents employed for accelerated aging cause the migration of the surfactant, favoring the capture of dust and causing mechanical-physical damages of the polymeric layer. For this reason, as introduced in the chapter “Experimental”, two cleaning practices were tested, in an attempt to evaluate the preliminary results by comparing their effectiveness on aged surfaces. The observations using 3D optical microscopy show how the two methods have a different cleaning impact depending on the type of gaseous pollutant used. In Figure S1, the acrylic films aged with RH 80% and H_2_S have a surface free of surfactant particles after the application of the gel. 

The samples aged with SO_2_ and NO_x_ show the same effect, if cleaned with cotton swab rolled, and finally, for the samples aged with O_3_ the cleaning action is the same with both practices. However, it is evident that the two cleaning methods have different cleaning agent release on the surface. Therefore, the water action causes mechanical-physical damages to the acrylic matrix (swelling). The AFM-Raman spectroscopy results will extend this behavior.

### 3.2. AFM Combined with Raman Spectroscopy

#### 3.2.1. After Aging

**Figure 2:**
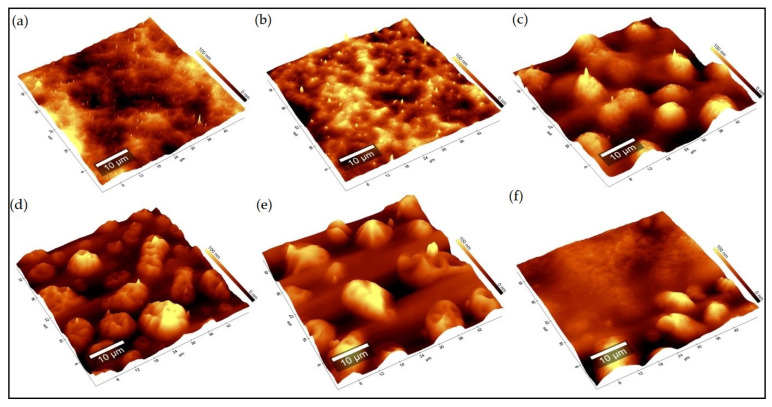
AFM topography images of acrylic films: (**a**) unaged; (**b**) RH 80%; (**c**) H_2_S; (**d**) SO_2_; (**e**) O_3_; (**f**) NO_x_ aged.

Acrylic surfaces exposed to accelerated gas aging were analyzed by AFM combined with Raman spectroscopy. Figure 2 shows the comparison between the topographies of the unaged sample (Figure 2a) and those of the samples aged with the single pollutants. As already observed, the high relative humidity used (RH 80%) caused the particle migration to the surface; however, the exposure to the various gaseous pollutants may determine a variable morphology and distribution of the amorphous particles. From the observation of the topographies and the evaluation of the roughness values (Sa) in Table 3, it is evident that all aging exposures result in a morphological change of the surface. However, it is observed that, with the same exposure time and relative humidity amount mixed with the gas, the degradation effects change. In fact, all pollutant gases favor the superficial migration of the particles based on their concentration, corrosive power, and solubility in humidified air [36]. Exposing the sample only to humidity, the morphological change mainly involves the acrylic component of the binder, resulting in swelling [37]. The different polymer/aging condition affinity is also verified by the different migration behaviors on the surface. In fact, they present a larger particle size when exposed to H_2_S and NO_x_ and more inhomogeneous distribution when exposed to O_3_ and NO_x_. Probably, this behavior is due to the easier dissolubility of NO_x_ in humidified water mixed in the weathering chamber and the chemical affinity between surfactant and NO_x_ [38]. 

**Figure 3:**
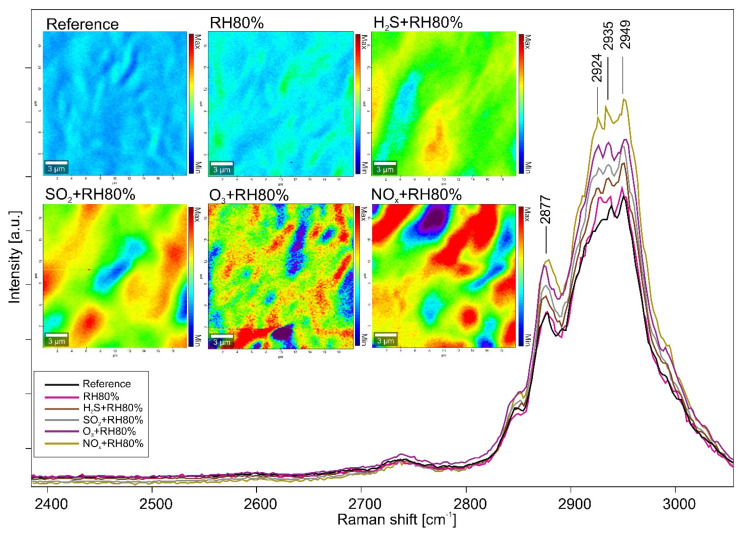
Raman chemical mapping of the PEO band at 2924 cm^−1^ for all investigated acrylic samples.

By combining AFM images with Raman chemical mappings (Figure 3), it was possible to identify the polymeric binder as methyl methacrylate and butyl acrylate compound (PMMA-nBA) by the presence of the main bands at 813 and 847 cm^−1^ of C-H rocking, 1300 cm^−1^ of C-H twisting/rocking, 1456 cm^−1^ of the C-H bending, 1738 cm^−1^ of the C=O stretching, and 2877, 2935, and 2950 cm^−1^ of the C-H stretching [39]. Furthermore, the band at 2924 cm^−1^ was identified as a surfactant signal, i.e., polyethylene oxide (PEO) [40]. After normalization of the spectra obtained from the scanned sections, the band area of the surfactant was integrated, and the chemical mapping for each aged sample was acquired. As shown with the AFM topographies, the impact of the pollutant gases on the surfaces favors the migration of the surfactant, which, depending on the gas exposure, assumes different conformations and distributions. The worst degrading effect is observed for the samples aged with NO_x_ and O_3_, subsequently decreasing with SO_2_, H_2_S, and finally RH 80%.

#### 3.2.2. After Cleaning

**Figure 4:**
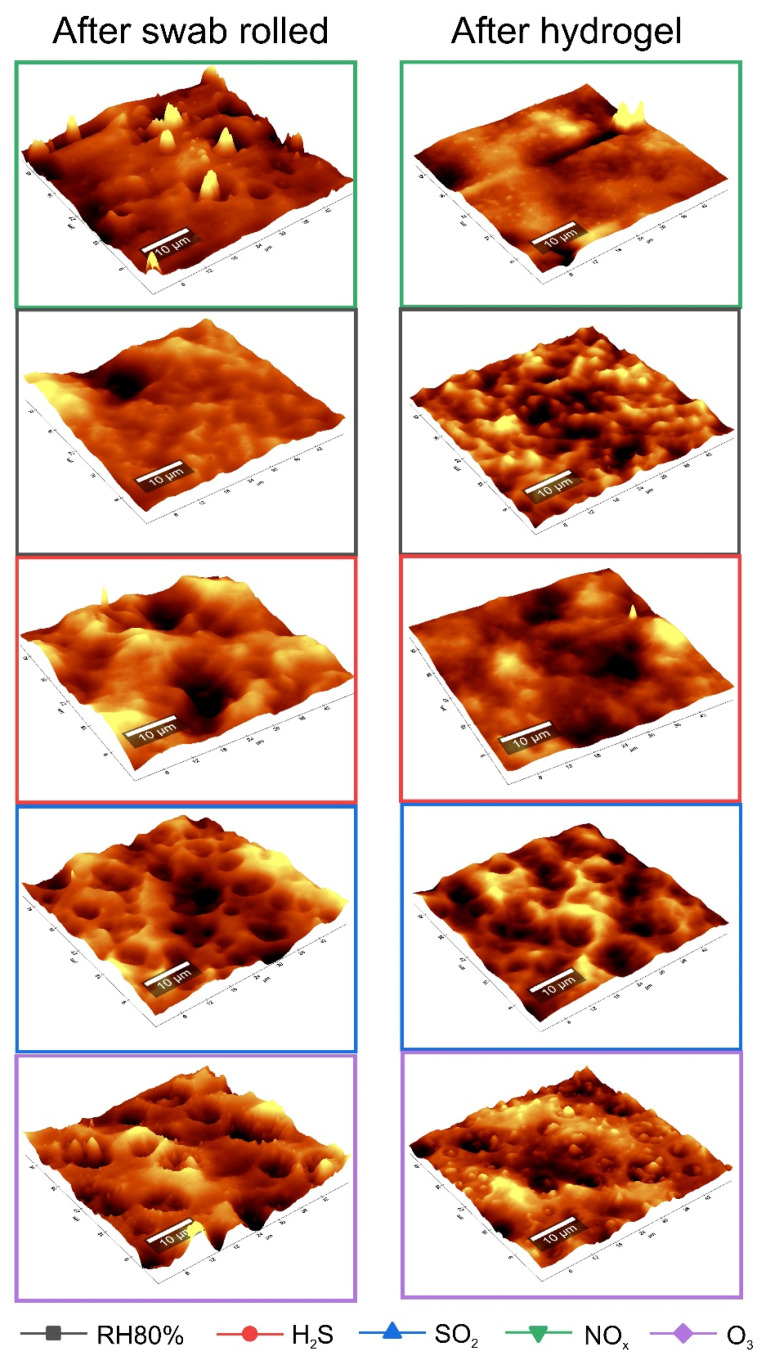
AFM topographies after gas aging and cleaning with swab rolled and hydrogel test.

The same data processing and morphological-chemical investigation were carried out to evaluate the cleaning method’s efficacy under examination [41]. Figure 4 and Table 4 compare the topographies and related roughness values (Sa) between the aged and cleaned samples using a cotton swab rolled and hydrogel test. In detail, the most effective cleaning method would result from the swab rolled test, obtaining Sa values very similar to those of the unaged acrylic surface. Using the hydrogel, these values remain high; however, if compared to the aged sample, they are lower. Furthermore, samples aged with NO_x_ appear to be less subject to cleaning, while the Sa value of the RH 80% aged sample slightly increases, probably due to the action of water that favored the swelling process. For samples cleaned with the swab rolled, the trend slightly changes. NO_x_ is still the pollutant causing the worst degradation effect; whereas, the surface exposed to O_3_, once cleaned, shows morphological changes comparable to those of unaged samples. On the other hand, observing the Raman chemical mappings, a superficial morphological improvement does not always correspond to a total removal of the surfactant particles on the surface. Integrating the PEO band (2924 cm^−1^), according to the different pollutant exposure, the effectiveness of the cleaning method changes. In samples aged with RH 80% and H_2_S, the surfactant particles easily dissolve when treated with the gel; those aged with SO_2_ and NO_x_ when treated with swab rolled, while those aged with O_3_ show a similar cleaning effect with both methods.

As previously mentioned for the 3D Optical Microscopy results, it was possible to determine with Raman chemical mappings that the action of the water had an impact on the acrylic matrix. In fact, with both cleaning methods, the release of the water on the surface led to the deformation of the polymeric film; moreover, despite the removal of the surfactant particles, they left cavities on the surface layer of the acrylic binder. These cavities could allow the accumulation of dirt, and favor the penetration of subsequent pollutants (UV radiation, pollutant gas, humidity, temperature), leading to further degradation processes [36].

### 3.3. ATR-FTIR Spectroscopy

#### 3.3.1. After Aging

**Figure 5:**
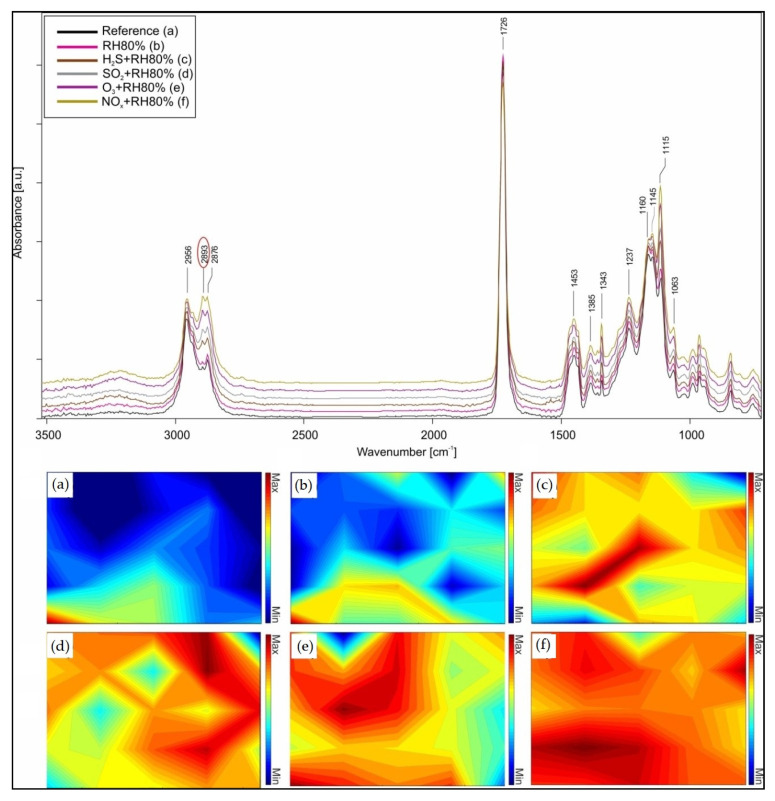
ATR-FTIR spectra and chemical mapping of the surfactant absorbance band integration at 2895 cm^−1^: (**a**) before aging and after (**b**) RH 80%, (**c**) H_2_S + RH 80%, (**d**) SO_2_ + RH 80%, (**e**) O_3_ + RH 80%, and (**f**) NO_x_ + RH 80%.

The main functional bands of acrylic samples were identified using ATR-FTIR analysis and listed in Table S2. The specific co-polymer identified is nBA/MMA through the following spectral IR signals: the stretching vibrations of the C-H bond (at 2956–2876 cm^−1^), the C=O stretching (at 1726 cm^−1^), and the bands of the C-O-C and C-O stretching (at 1236, 1160, 1146 cm^−1^) in the fingerprint region [33]. Furthermore, the three absorbance bands at 2895, 1343, and 1115 cm^−1^ were characterized as spectral signals of the surfactant PEO (polyethylene oxide) [42,43], confirming the previous Raman characterization. To understand the different degradation processes of the acrylic binder, FTIR chemical mapping was performed. It was used to investigate the various surface distributions of the surfactant according to the different pollutants and compared the results with Raman ones. The surfactant spectral signal (2895 cm^−1^) was integrated for the mapping of the acrylic emulsions, and its distribution on the scanned area was evaluated. This absorbance band was integrated to better compare the chemical mapping performed with Raman spectroscopy in which the same functional group was integrated. In Figure 5, it is observed that the inhomogeneous surfactant distribution on the surface is mainly favored by NO_x_, followed by O_3_, SO_2_, H_2_S, and finally RH 80%, confirming the previous Raman results. They also lead to the understanding that surfactant particles differ not only morphologically but also by molecular analysis. In fact, the intensity of the integrated absorbance band appears to be higher for the samples aged with NO_x_ and O_3_, confirming that the surfactant particles are more widespread and thicker under these aging conditions (as observed with the AFM evaluation of roughness).

To support this evaluation, semi-quantitative analysis was performed by integrating the three spectral bands of the surfactant (2895, 1343, 1115 cm^−1^) and comparing the values obtained from the unaged and aged samples. In Figure S2 and Table S3, the degradation trends and the respective plotted values are presented. From this evaluation, it is possible to understand that the deteriorating effect is more significant with NO_x_ than with other pollutant gases. It seems that the band at 1115 cm^−1^ is more affected, contrary to the other two bands; however, the corrosive trend (NO_x_ > O_3_ > SO_2_ > H_2_S > RH80%) is always observed. Additionally, the integration of the band at 1726 cm^−1^ of the carbonyl group (acrylic component) and the calculation of the difference between these values and those of the band at 1115 cm^−1^ were performed. It is observed that the increase of the IR signal of the surfactant is directly proportional to the decrease of the carbonyl band, indicating that the pollutants not only favor the migration of the surfactant but also oxidize the functional groups of the polymeric matrix.

These degradation mechanisms can be explained by considering the solubility kinetics of the various pollutant gases used. As underlined by the results presented, NO_x_ and O_3_ are the most impacting corrosive agents on the stability of the acrylic emulsions, although they have two different physical-chemical behaviors in an aqueous environment. The solubility of NO_x_ is the least understood, as it has numerous species and reactions involved in the reactive process [44]. In this study, the gaseous mixture used mainly includes two nitrogen oxides, namely NO and NO_2_. According to the literature [38,45], NO (nitric oxide) hardly dissolves in water, while NO_2_ (nitrogen dioxide) reacts immediately with water upon dissolution resulting in HNO_3_ (nitric acid). It leads to a stronger corrosive kinetics than the other pollutant gases causing bond breaking of the acrylic matrix by oxidation and the promotion of surfactant migration to the surface. Furthermore, as shown by previous studies [46], NO_x_ has a greater affinity with organic compounds that, summed to the capability to dissociate in an aqueous environment easily, promotes hydrolysis reactions. It leads to an increasing level of degradation of acrylic films, also depending on the relative humidity value in the environment. On the contrary, when O_3_ is in an aqueous environment (or at high humidity values), it leads to the formation of radical species [47]. These radicals are strong oxidizing agents attacking unsaturated linkages of the acrylic emulsions. Furthermore, the presence of water causes hydrolysis reactions attacking the sensitive groups present in the polymer. Specifically, PBMA is not one of the most stable conformations of acrylic emulsion and, if exposed to deteriorating agents, it undergoes a rapid and extensive fragmentation, leading to an overall loss of structural and molecular properties [48].

#### 3.3.2. After Cleaning

**Figure 6:**
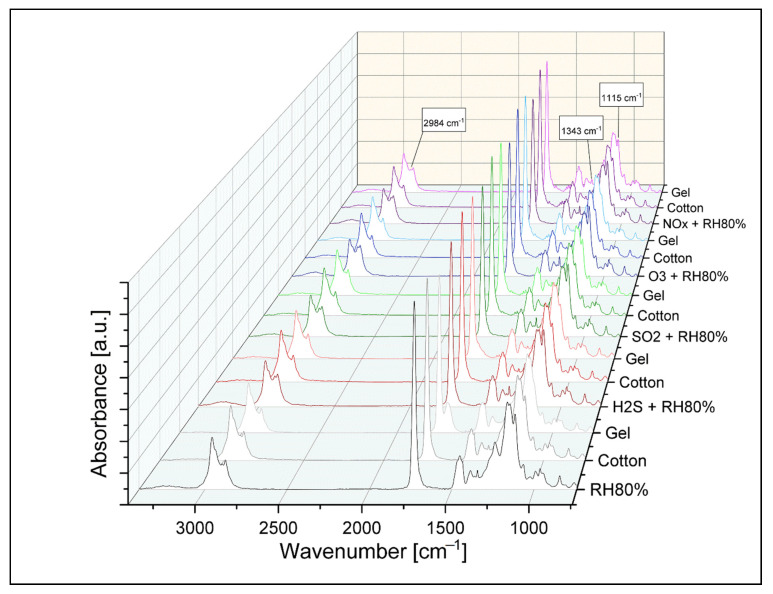
Comparison between ATR-FTIR spectra of aged samples and their surfaces after cotton swab rolling and gel test.

An ATR-FTIR evaluation was also performed after the subsequent cleaning treatments of the acrylic emulsions. It was important to understand the cleaning level of the two chosen methods (swab rolled and hydrogel test) both at a qualitative and semi-quantitative level. As shown in Figure 6, both cleaning procedures reduce the intensity some spectral signals characterized as surfactant bands. In particular, the signal at 1115 cm^−1^ verifies the impact of the different releases of water on the surface, according to the chosen degradation conditions. In fact, supporting these results with the FTIR chemical mappings carried out on larger areas (Figure S3 and Table S4), it is evident how the surface particles of surfactant are reduced. In particular, the use of the swab rolled test favors the cleaning of acrylic surfaces aged by SO_2_ and NO_x_, whereas the hydrogel test is more effective on those aged by RH 80% and H_2_S. Both cleaning methods on O_3_ aged samples are equally suitable.

However, as mentioned in the literature [49,50], the aqueous treatments always cause physical-mechanical damage on acrylic emulsions. The two methods proposed have advantages as well as disadvantages. Although the swab rolled is the most effective, as it allows roughness values similar to the initial ones to be reached, it causes more significant surface swelling effects. In fact, the water release on the surface is not easily controlled, and the direct contact intensified by mechanical action causes damages to the acrylic matrix, making the film structurally and chemically weaker and prone to the attack of subsequent pollutants (dust, dirt, light radiation, humidity, air pollutants).

On the other hand, the hydrogel can modulate the aqueous impact on the acrylic surface (less swelling), but the cleaning action seems to be more effective on surfaces subject to high humidity values or on aged samples with less aggressive pollutant gases. This issue may be solved by increasing the time of gel application on the acrylic emulsions, while continuing to monitor possible structural changes. Finally, in some regions of samples, an ATR-FTIR band at 1680 cm^−1^ was observed after the application of the gel. It was identified as gel residue (Figure 6) [51].

## 4. Conclusions

In this study, accelerated aging by pollutant gases and relative humidity of acrylic emulsion films was performed. The innovative aspect of this evaluation is the use of microscopic analyses not focusing only on the surface morphological observations but applying statistical evaluations to understand the particle size, distribution, and roughness of the degradation products according to the different pollutant gas employed. In addition to being compared with each other, the results will be implemented using non-invasive spectroscopic techniques that explain the chemical-physical nature of the degradation processes focused on the acrylic film surfaces. Another new aspect of this study is the comparative investigation between the results obtained after accelerated aging and those after cleaning. In detail, in the first part, the results focus on the characterization of acrylic emulsions and degradation products resulting from gaseous aging. The identification of this polymeric system is complex, as some molecular structures are very similar, and some formulations include the presence of different additives. In this specific study, polyethylene oxide (PEO) was identified as the main additive in the acrylic emulsions. As it has hygroscopic properties, once it is exposed to certain aging conditions, it migrates to the surface in the form of particles which, from observations to the 3D microscope, cause the opacification of the superficial acrylic component. This behavior can influence the mechanical resistance, the adhesion, the permeability, and the attraction of dirt on the acrylic emulsion surfaces. Furthermore, depending on the type of pollutant, different changes concerning the superficial distribution, particle size, and roughness are observed. From the evaluation and comparison of the microscopic (3D and atomic force microscopy) and chemical (Raman and FTIR spectroscopy) results, the gaseous pollutant that causes the greatest damage to acrylic emulsions, in terms of structural impact, increases of roughness values, and extends the dispersion of surfactant particles is NO_x_, followed by O_3_, SO_2_, H_2_S, and RH 80%. Probably, this phenomenon is related to the solubility of gases in humidified environment and the chemical surfactant–gas affinity.

Subsequently, the study focused on the preliminary evaluation of the effectiveness of some cleaning treatments used for the surfactant removal. The choice of the most appropriate cleaning method for acrylic emulsions is a topic still discussed today because, depending on the treatment used, it can damage the structural and chemical integrity of the acrylic matrix. For this reason, two cleaning procedures were considered: the first using cotton swab rolled and the second using a hydrogel system. From the assessments performed, both treatments have advantages and disadvantages. From the AFM topographies and chemical mappings, surfactant removal is more effective with cotton swab rolled; however, it compromises the structural stability of the acrylic component by causing surface swelling. On the other hand, although the hydrogel allows the gradual release of water for a more controlled cleaning and limits swelling, it does not allow a regular cleaning effect and gel residues are observed after application. The cleaning effects are also influenced by the interaction between acrylic film and pollutant; in fact, the cotton swab rolled test is more effective on samples aged by SO_2_ and NO_x_, whereas the hydrogel on those aged by RH80% and H_2_S.

The results obtained so far show how various physical-chemical interactions are observed, depending on the formulations of the acrylic emulsions and the influence of the gaseous pollutant agents. The results obtained from the mock-up tests could then be confirmed and expanded with further investigations (on real cases, commercial acrylic paints, use of different solvents for cleaning, additional accelerated aging conditions) and analytical-diagnostic analysis (mechanical, thermal, separation techniques). The study of the deteriorating behaviors deriving from the gaseous exposure of acrylic paints, mixed with different inorganic and organic pigments, could be a further research topic as they can promote or reduce the consequent degradation reactions [52,53]. In addition to the use of different solvents for cleaning acrylic surfaces, a further implementation of the study can be to test new cleaning products and monitor their effectiveness on the surfaces [54]. 

To extend the knowledge about artificial gas aging, future experiments will be considered. As mentioned in the introduction, the effect of different pollutant gases and particulate matter also significantly influences the stability of artworks. Therefore, a future study can be the cross evaluation of pollutants. The combination of two or more pollutants and their synergy with different relative humidity values, gas concentration, and exposure time, will provide additional information about their chemical stability and the oxidizing effect. Furthermore, this study provides further clarifications regarding some conservative aspects highlighted in the museum environment [55], related to the degradation processes deriving from environmental variables, and the effectiveness of cleaning treatments on aged acrylic objects.

## Figures and Tables

**Table 1:** Gas selected for the artificial accelerated aging and corresponding concentrations chosen.

Gas Pollutant	Concentration (ppm)	Relative Humidity (RH%)
H_2_S	0.25	80
SO_2_	15	80
NO_x_	15	80
O_3_	2500	80

**Table 2:** Parameters derived from the particle size histogram corresponding to Gaussian equations.

Samples	Weathering Conditions	Average Particle Size (μ)	Standard Deviation (σ)	Correlation Coefficient (R)
Acrylic emulsion films	H_2_S + RH80%	8.35 µm	±2.09 µm	0.92
	SO_2_ + RH80%	6.84 µm	±2.93 µm	0.91
	O_3_ + RH80%	8.38 µm	±3.28 µm	0.98
	NO_x_ + RH80%	10.56 µm	±6.5 µm	0.99

**Table 3:** AFM roughness values for acrylic films.

Samples	Weathering Conditions	Sa [nm]	Average Particle Size [µm]
Pure acrylic film	Unaged	76.3	-
	RH80%, 168 h	95.2	-
	H_2_S, RH80%, 168 h	207.5	8.74 ± 1.24
	SO_2_, RH80%, 168 h	231.6	5.44 ± 2.1
	O_3_, RH80%, 168 h	333.5	7.84 ± 4.5
	NO_x_, RH80%, 168 h	407.8	10.8 ± 6.7

**Table 4:** AFM roughness values (Sa) obtained after gas aging and after cleaning treatments.

Weathering Conditions	Sa [nm]		
	After Aging	After Swab Rolled	After Hydrogel
RH 80%	95.1	85.2	123.5
H_2_S + RH 80%	207.4	75.9	145.7
SO_2_ + RH 80%	231.6	75	136
O_3_ + RH 80%	333.4	62.5	146.6
NO_x_ + RH 80%	407.8	174.1	385.7

## Data Availability

Additional data about 3D images, ATR topographies, Raman and IR results and chemical mappings are available upon request.

## Supplementary Materials

The following are available online at https://www.mdpi.com/article/10.3390/polym13121941/s1. Figure S1. Microscopic images of pure acrylic binder before and after cleaning. From the left, the aged surfaces are shown according to gas aging. On the right, the cleaned surfaces after swab rolled test and hydrogel application. Figure S2. Semi-quantification evaluation of selected spectral signals at 2895, 1343, 1115 cm^−1^ divided by five different pollutant aging. To the initial integration area (unaged samples), the difference between the unaged and aged were added for each set of sample. Figure S3. Chemical mapping of surfactant band at 1115 cm^−1^ after swab rolled and hydrogel tests on all aged samples. Table S1. Integrated bands for semi-quantification evaluation of surfactant migration. Table S2. ATR-FTIR band assignment of acrylic emulsion films analyzed. Table S3. Integrated area values of acrylic samples analyzed corresponding to Figure S2. Table S4. Integrated area values of surfactant IR signal after cleaning.
