# Supplementary material for: The Effect of Pollutant Gases on Surfactant Migration in Acrylic Emulsion Films: A Comparative Study and Preliminary Evaluation of Surface Cleaning
Source: Polymers (Basel). 2021 Jun 11;13(12):1941. doi: 10.3390/polym13121941 (PMC8230686; doi:10.3390/polym13121941)
Supplement: Supplementary file 1 [file polymers-13-01941-s001.zip › polymers-1247047-supplementary.pdf]

SUPPLEMENTARY MATERIAL

The effect of pollutant gases on surfactant migration in acrylic emulsion films: a comparative study and preliminary evaluation of surface cleaning

Laura Pagnin[1*], Rita Wiesinger[1], Ayse Nur Koyun[2], Manfred Schreiner[1,3]

[1]*Academy of Fine Arts Vienna, Institute of Science and Technology in Art, Schillerplatz 3, 1010 Vienna, Austria*
[2]*Technische Universität Wien, Institute of Materials Chemistry, Getreidemarkt 9/165, 1060 Vienna, Austria*
[3]*Technische Universität Wien, Institute of Chemical Technologies and Analytics, Getreidemarkt 9/164, 1060 Vienna, Austria*

*Email: l.pagnin@akbild.ac.at

Table of Contents

Table S1. Integrated bands for semi-quantification evaluation of surfactant migration. 2

Figure S1 Microscopic images of pure acrylic binder before and after cleaning. From the left, the aged surfaces are shown according to gas aging. On the right, the cleaned surfaces after swab rolled test and hydrogel application. 2

Table S2. ATR-FTIR band assignment of acrylic emulsion films analyzed. 3

Figure S2. Semi-quantification evaluation of selected spectral signals at 2895, 1343, 1115 cm^{-1} divided by five different pollutant aging. To the initial integration area (unaged samples), the difference between the unaged and aged were added for each set of sample. 3

Table S3. Integrated area values of acrylic samples analyzed corresponding to Figure S2. 4

Table S4. Integrated area values of surfactant IR signal after cleaning. 4

Figure S3. Chemical mapping of surfactant band at 1115 cm-1 after swab rolled and hydrogel tests on all aged samples. 5

Table S1. Integrated bands for semi-quantification evaluation of surfactant migration.

Sample	Integrated band [cm^{-1}]	Corresponding range [cm^{-1}]	Assignment
Acrylic binder	2894	2904-2883	Polyethylene oxide signal
	1343	1351-1334	Polyethylene oxide signal
	1115	1126-1100	Polyethylene oxide signal

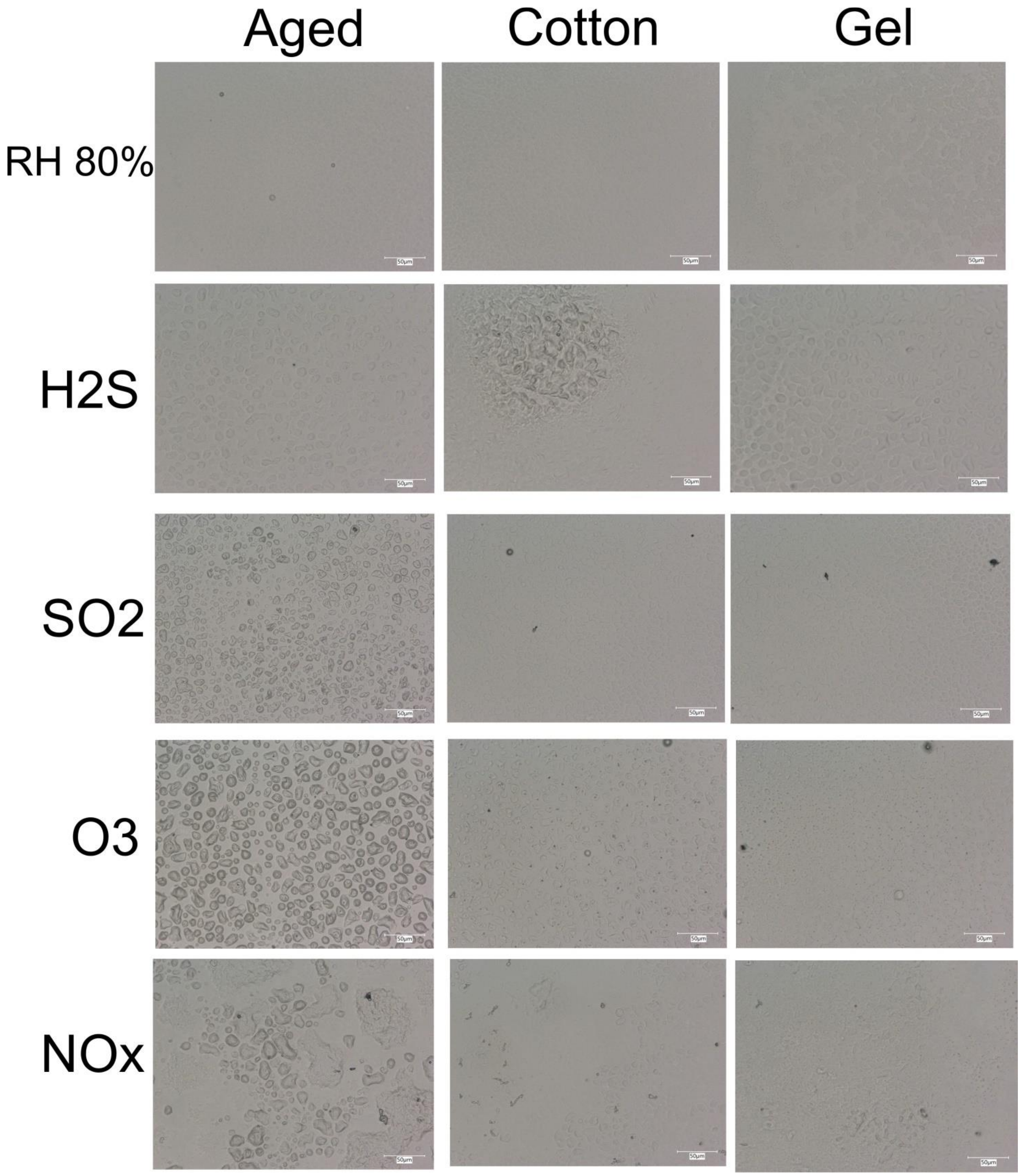

Figure S1. Microscopic images of pure acrylic binder before and after cleaning. From the left, the aged surfaces are shown according to gas aging. On the right, the cleaned surfaces after swab rolled test and hydrogel application.

Table S2. ATR-FTIR band assignment of acrylic emulsion films analyzed.

Samples	Wavenumber [cm^{-1}]	Absorption band assignment
Acrylic emulsion films	2956 – 2876	C-H stretching (sym-asym)
	2895	*Polyethylene oxide (PEO)*
	1726	C=O stretching
	1450 – 1385	CH_3-CO- (asym)
	1343	*Polyethylene oxide (PEO)*
	1236	C-O-C stretching
	1160-1146	C-O stretching
	1115	*Polyethylene oxide (PEO)*
	1064	C-O stretching
	989-963	C-C stretching
	843	C-H rocking

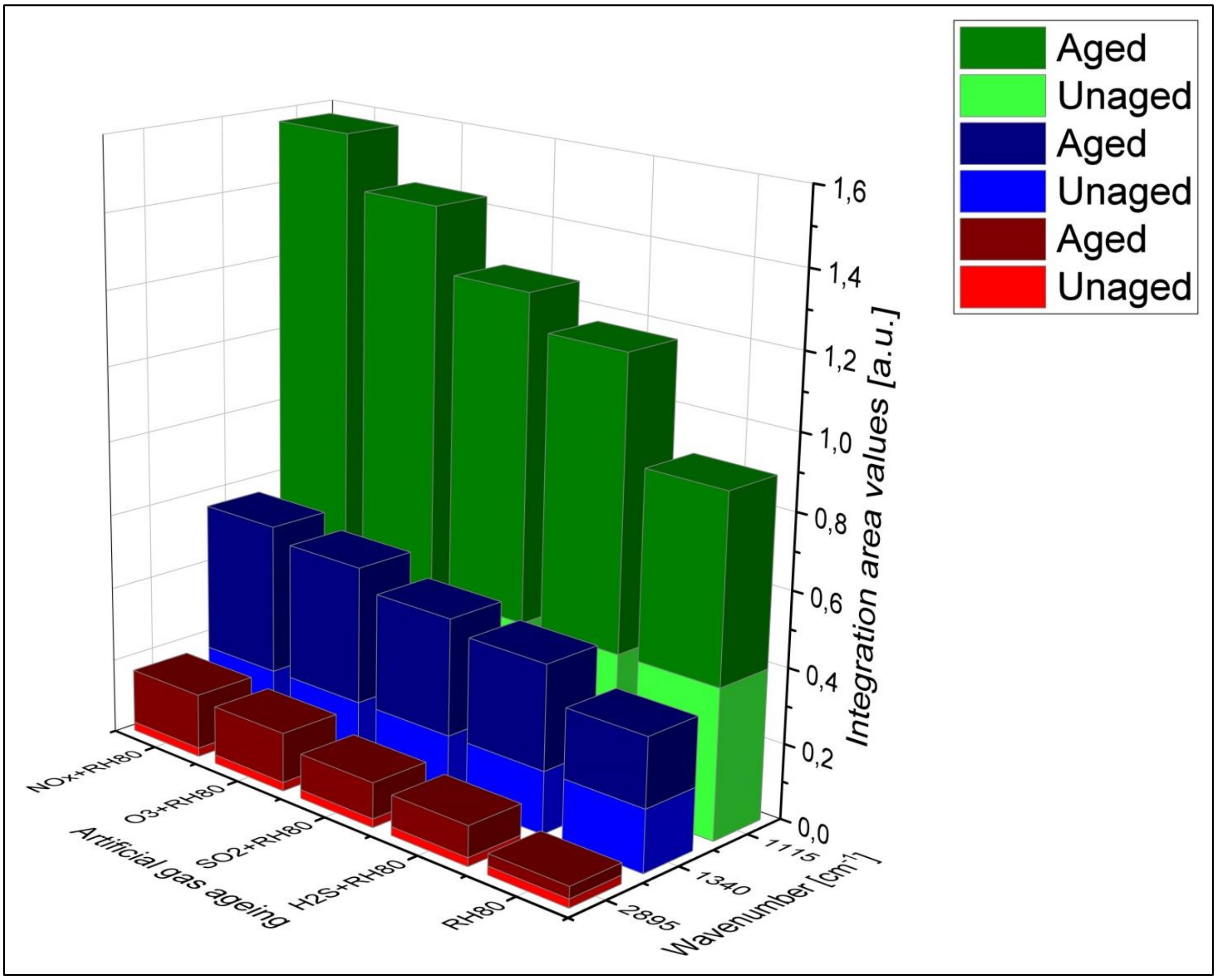

Figure S2. Semi-quantification evaluation of selected spectral signals at 2895, 1343, 1115 cm^{-1} divided by five different pollutant gas aging conditions. To the initial integration area (unaged samples), the difference between the unaged and aged were added for each set of sample.

Table S3. Integrated area values of IR absorbance bands of acrylic samples analyzed.

Sample	Accelerated aging condition	Integrated area for band at 2895 cm^{-1}			Integrated area for band at 1340 cm^{-1}		
		**Unaged**	**Aged**	**Sum integrated values**	**Unaged**	**Aged**	**Sum integrated values**
Acrylic emulsion film	RH80%	0.023	0.032	0.055	0.166	0.184	0.35
	H_2S + RH80%	0.023	0.083	0.106	0.166	0.278	0.444
	SO_2 + RH80%	0.023	0.096	0.119	0.166	0.311	0.477
	O_3 + RH80%	0.023	0.133	0.156	0.166	0.366	0.532
	NO_x + RH80%	0.023	0.147	0.17	0.166	0.401	0.567
		**Integrated area for band at 1115 cm^{-1}**			**Integrated area for band at 1726 cm^{-1}**		
		**Unaged**	**Aged**	**Sum integrated values**	**Unaged**	**Aged**	**Difference***
	RH80%	0.399	0.495	0.894	17.00	17.288	**16.793**
	H_2S + RH80%	0.399	0.771	1.17	17.00	16.95	**16.179**
	SO_2 + RH80%	0.399	0.863	1.262	17.00	16.272	**15.409**
	O_3 + RH80%	0.399	1.031	1.43	17.00	16.079	**15.048**
	NO_x + RH80%	0.399	1.173	1.572	17.00	15.141	**13.968**

*Difference between integrated area values of band at 1726 cm^{-1} and band at 1115 cm^{-1} of aged samples.

Table S4. Integrated area values of surfactant IR signal after cleaning.

Sample	Accelerated aging condition	Integrated area for band at 1115 cm^{-1}		
		**Aged**	**Swab rolled**	**Gel**
Acrylic emulsion film	RH80%	0.495	0.500	**0.081**
	H_2S + RH80%	0.771	0.453	**0.228**
	SO_2 + RH80%	0.863	**0.398**	0.581

	O_3 + RH80%	1.031	0.381	0.357
	NO_x + RH80%	1.173	**0.553**	0.771

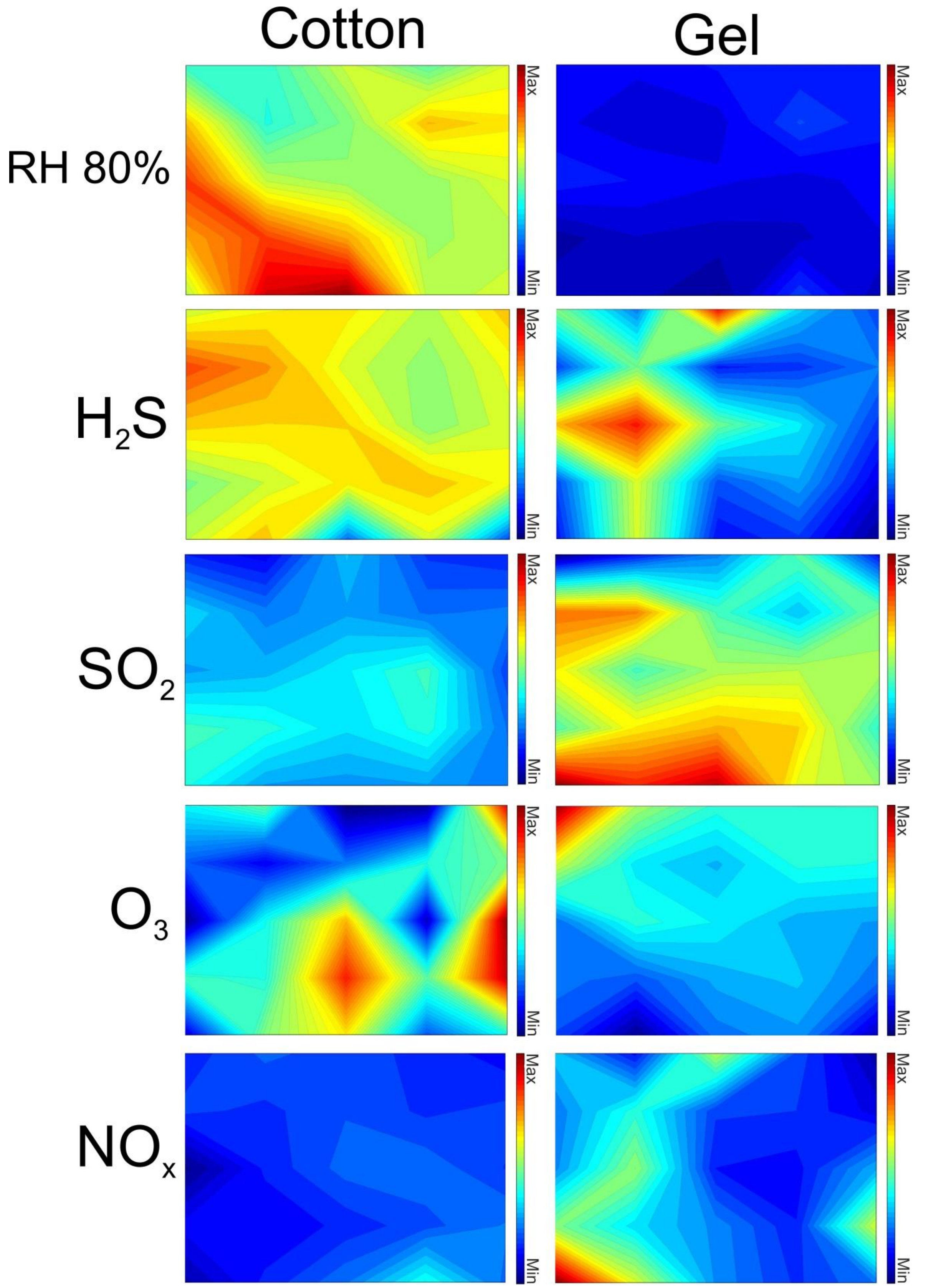

Figure S3. Chemical mapping (1.0 x 1.5 mm^2) of surfactant band at 1115 cm^{-1} after swab rolled and hydrogel tests on all aged samples.
